# Critical Closing Pressure and Cerebrovascular Resistance Responses to Intracranial Pressure Variations in Neurocritical Patients

**DOI:** 10.1007/s12028-023-01691-8

**Published:** 2023-03-03

**Authors:** Sérgio Brasil, Ricardo de Carvalho Nogueira, Ângela Salomão Macedo Salinet, Márcia Harumy Yoshikawa, Manoel Jacobsen Teixeira, Wellingson Paiva, Luiz Marcelo Sá Malbouisson, Edson Bor-Seng-Shu, Ronney B. Panerai

**Affiliations:** 1 Division of Neurosurgery, Department of Neurology, School of Medicine, University of São Paulo, Av. Dr. Eneas de Carvalho Aguiar 255, São Paulo, Brazil; 2 Department of Intensive Care, School of Medicine, University of São Paulo, São Paulo, Brazil; 3 Department of Cardiovascular Sciences, School of Life Sciences, University of Leicester, Leicester, UK; 4 National Institute for Health and Care Research, Cardiovascular Research Centre, Glenfield Hospital, University of Leicester, Leicester, UK

**Keywords:** Cerebral perfusion pressure, Intracranial pressure, Intracranial compliance, Resistance-area product, Critical closing pressure, Acute brain injury

## Abstract

**Background:**

Critical closing pressure (CrCP) and resistance-area product (RAP) have been conceived as compasses to optimize cerebral perfusion pressure (CPP) and monitor cerebrovascular resistance, respectively. However, for patients with acute brain injury (ABI), the impact of intracranial pressure (ICP) variability on these variables is poorly understood. The present study evaluates the effects of a controlled ICP variation on CrCP and RAP among patients with ABI.

**Methods:**

Consecutive neurocritical patients with ICP monitoring were included along with transcranial Doppler and invasive arterial blood pressure monitoring. Internal jugular veins compression was performed for 60 s for the elevation of intracranial blood volume and ICP. Patients were separated in groups according to previous intracranial hypertension severity, with either no skull opening (Sk1), neurosurgical mass lesions evacuation, or decompressive craniectomy (DC) (patients with DC [Sk3]).

**Results:**

Among 98 included patients, the correlation between change (Δ) in ICP and the corresponding ΔCrCP was strong (group Sk1 *r* = 0.643 [*p* = 0.0007], group with neurosurgical mass lesions evacuation *r* = 0.732 [*p* < 0.0001], and group Sk3 *r* = 0.580 [*p* = 0.003], respectively). Patients from group Sk3 presented a significantly higher ΔRAP (*p* = 0.005); however, for this group, a higher response in mean arterial pressure (change in mean arterial pressure *p* = 0.034) was observed. Exclusively, group Sk1 disclosed reduction in ICP before internal jugular veins compression withholding.

**Conclusions:**

This study elucidates that CrCP reliably changes in accordance with ICP, being useful to indicate ideal CPP in neurocritical settings. In the early days after DC, cerebrovascular resistance seems to remain elevated, despite exacerbated arterial blood pressure responses in efforts to maintain CPP stable. Patients with ABI with no need of surgical procedures appear to remain with more effective ICP compensatory mechanisms when compared with those who underwent neurosurgical interventions.

**Supplementary Information:**

The online version contains supplementary material available at 10.1007/s12028-023-01691-8.

## Introduction

The interaction between the brain tissue, the cerebrospinal fluid (CSF), and the intracranial blood volume, inside an almost inexpansible skull [1], are the determinants of the intracranial pressure (ICP) [2]. ICP is the driving force that, in combination with the brain vascular network reactivity, especially to arterial pressure and CO_2_ changes, regulate cerebrovascular resistance (CVR) [3]. It is known that the counterforce between mean arterial blood pressure (MAP) and mean ICP indicates the resulting pressure with which the brain tissue will be perfused, known as cerebral perfusion pressure (CPP) [4]. In the case of acute brain injury (ABI), this relationship may not remain as accurate as for healthy study participants because of cerebrovascular autoregulation (CA) impairment [5, 6].

Acute brain damages related to traumatic brain injury, subarachnoid hemorrhage, and both hemorrhagic or ischemic strokes lead to either diffuse or focal injuries that rationally would conduce to a nonhomogeneous compromise of CPP, challenging the veracity of CPP remaining exclusively on the interactions of MAP and ICP [7]. Furthermore, severely ill patients receive arterial blood pressure (ABP) monitoring derived from a brachial arterial line that may overestimate CPP by up to 40 mm Hg, as central arteries handle lower resistance and pressures than peripheral arteries [8–10].

Therefore, assessment of cerebral hemodynamics is crucial for the neurocritical patient because ignoring it may lead to ventilatory, volemic, and pressure mismanagement [11]. Transcranial Doppler (TCD) is one of the most useful tools that can be employed at the bedside to assess cerebral hemodynamics by means of measurements of cerebral blood velocities (CBvs). Despite its excellent temporal resolution and usefulness to perform a number of critical diagnoses, the potential of TCD could be extended even further by providing beat-to-beat estimates of critical closing pressure (CrCP), the value of MAP with which capillary arteries blood flow stops [7, 12] and resistance-area product (RAP) [13, 14]. CrCP indicates the MAP value where cerebral blood flow reaches zero, which has been consistently shown to be well above 0 mm Hg in the cerebral circulation [7, 15]. RAP represents the slope of the instantaneous pressure–velocity relationship for each cardiac cycle [16, 17]. Although it could be seen as an alternative estimation of the CVR, the analogy is not entirely rigorous due to the presence of CrCP [16]. Because the brain capillaries and venules are more easily collapsible than the large vessels, not being observed in conventional tomographies and TCD examinations, CrCP and RAP have the potential to transmit more precise information about the impact of ICP over the microcirculation [18].

It is important to highlight that the brain is located within a rigid skull structure known as a fragile environment for changes in pressures as complications following ABI. Hence, the present study tests the hypothesis that changes in CrCP and RAP are strongly associated with changes in ICP in patients with ABI.

## Methods

This is a single center, prospective, observational study performed at Hospital das Clínicas, São Paulo University, Brazil. The study protocol was approved by the local Ethics Committee, in May/23/2017 (REB register 66721217.0.0000.0068) and registered under number NCT03144219 (available at ClinicalTrials.gov). All methods were performed in accordance with the relevant guidelines and regulations, and informed consent was obtained from all legally authorized representatives or next of kin instead of the patients because of illness severity.

### Participants and Protocol

The inclusion criteria were patients with a diagnosis of ABI within 5 days of hospital admission submitted to ICP monitoring according to the guidelines of the Brain Trauma Foundation for patients with traumatic brain injury and tomographic evaluation in the case of nontraumatic patients. Exclusion criteria were absence of TCD acoustic windows, neurological examination indicative of brain death, and absence of informed consent. Computed tomography (CT) scans, performed within 24 h prior or posterior to inclusion, were assessed to separate patients in groups according to injury severity, corresponding to the following: no skull opening group (Sk1), surgical mass evacuation group (Sk2), and primary decompressive craniectomy (DC) group (Sk3). Decision for surgical management was performed according to the local institutional neurosurgical guidelines. Nevertheless, surgical management was based either on the presence of mass lesions greater than 30 cm^3^, midline shift more than 0.5 cm, or imminent risk of herniation in the case of severe brain swelling on admission CT scans. Clinical and laboratory variables were collected at the day of study inclusion.

### Physiological Measurements

CBv was recorded bilaterally in the middle cerebral arteries (MCAs) with TCD (Doppler Box; DWL Compumedics, Singen, Germany) equipped with a 2-MHz probe. Recording was initiated after the best MCA signal quality was acquired and without insonation angle variation during the session. Intraventricular ICP was measured with the Neurovent monitoring system using a solid-state transducer (Raumedic®, Munchberg, Germany). ABP was recorded invasively with a radial artery catheter. The pressure transducer was leveled and zeroed to the intersection of the anterior axillary line and the fifth intercostal space. End-tidal CO_2_ (EtCO_2_) was measured continuously with an infrared capnograph (Dixtal, DX 1265 EtCO_2_; Capnogard, Manaus, Brazil). Prior to data collection, an ultrasound examination was performed to discard significant intracranial stenosis [19]. Monitoring was performed for 10 min at rest; at minute 7, an ultrasound guided (Sonosite Micromaxx 13 MHz, USA) sudden manual bilateral internal jugular veins compression (IJVC) was performed for 60 s. The U.S. guidance was used to standardize the compression technique for all patients and to ensure no compression of the carotid arteries. For patients with intracranial hypertension, the IJVC was precluded or aborted in the case of dampened CBv verified prior or during the maneuver [20]. Continuous and simultaneous measurements of CBv, ABP, and ICP were integrated by using the analog-to-digital converter of the Doppler Box at 100 samples/s and stored for off-line editing and analysis.

### Data Editing

Beat-to-beat data were analyzed by using in-house custom software written in Fortran. Continuous recordings were visually inspected and narrow artifacts (< 100 ms) were removed by linear interpolation. Spikes in the CBv channels were removed with a median filter and all signals were low-pass filtered with a zero-phase eighth-order Butterworth filter with a cut-off frequency of 20 Hz. The beginning and end of each cardiac cycle was detected in the ABP signal, visually checked, and used to obtain beat-to-beat values of heart rate, MAP, mean CBv, and ICP. The instantaneous relationship between ABP and CBv was used to estimate CrCP and RAP for each cardiac cycle using the first harmonic method [7]. Beat-to-beat data were spline interpolated and resampled at 5 Hz to produce signals with a uniform time base*.*

### Data Analysis

**Fig. 1:**
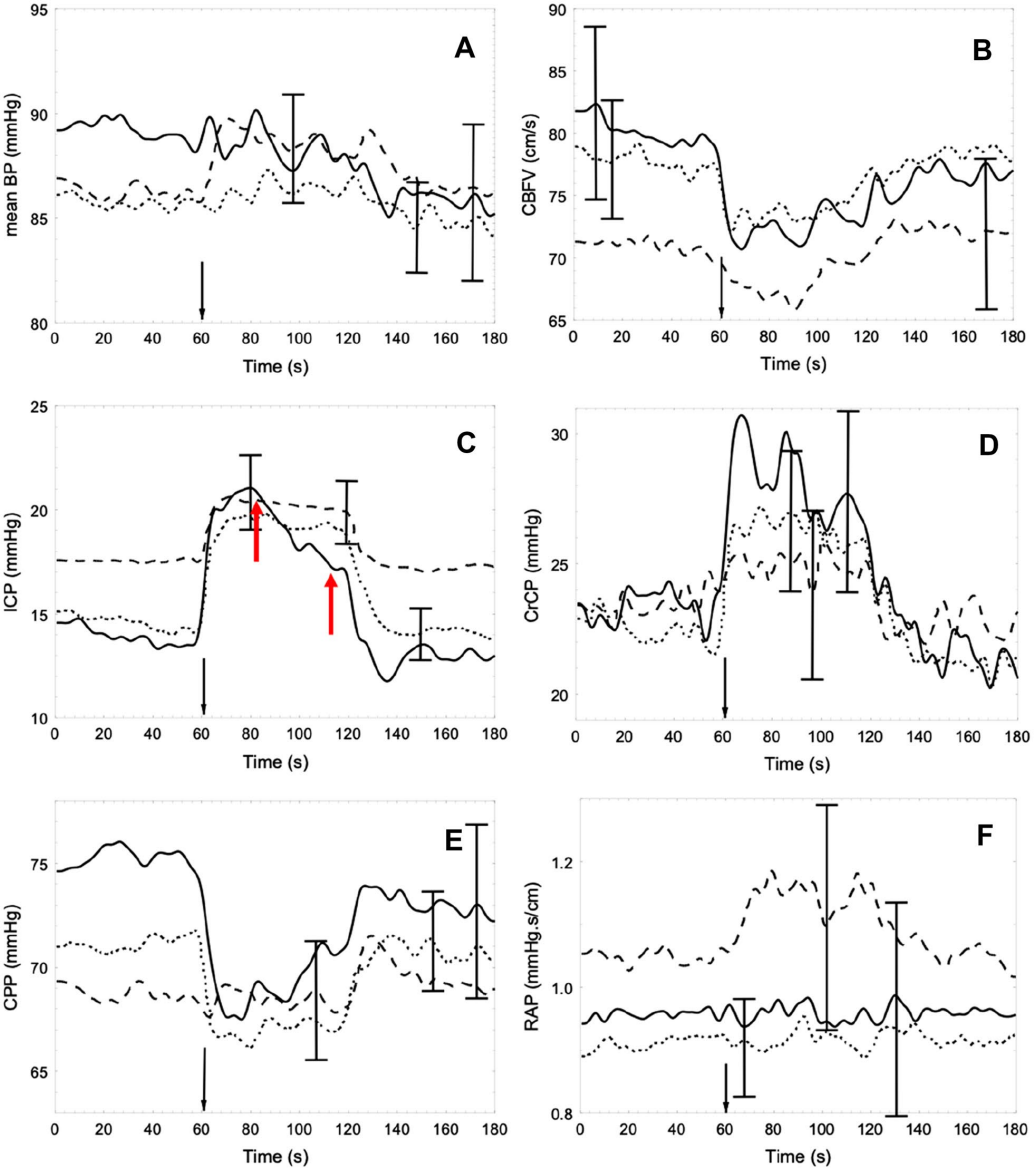
Population average of cerebral hemodynamic parameters following temporary compression of the internal jugular vein in patients with traumatic brain injury. The vertical black arrow marks the beginning of compression. **a** Mean arterial blood pressure (MAP); **b** cerebral blood velocity; **c** intracranial pressure (ICP), with notably spontaneous ICP lowering after 20 s of IJVC (interval between red arrows) exclusively for group Sk1; **d** critical closing pressure (CrCP); **e** cerebral perfusion pressure (CPP) (CPP = MAP − ICP); and **f** resistance-area product (RAP). Representation corresponds to group Sk1 (undamaged skull, continuous line, *n* = 23), group Sk2 (craniotomy, dotted line, *n* = 35), and group Sk3 (decompressive craniectomy, dashed line, *n* = 22). The error bars represent ± 1 SE at the time of occurrence. BP, blood pressure; CBFV, cerebral blood flow velocity; IJVC, internal jugular veins compression; SE, standard error of the mean

The resampled time-series of ICP was visually inspected, and the beginning of compression was marked. All recorded variables were then synchronized with the instant of compression placed at time = 60 s within a 3-min data window (Fig. 1), and the population coherent average and standard deviation were obtained for each variable (MAP, ICP, CPP, CBv, CrCP, and RAP) for each of the skull condition groups. Patients of any age and sex were included, from children to older people, although their influence on results was analyzed separately. Baseline values were calculated as the mean for the first 60 s, before the beginning of compression. Mean values calculated for the first 30 s of compression were used with baseline values to obtain the change (Δ) in each variable due to compression.

### Statistical Analysis

Parameter distributions were expressed as mean ± standard deviation. The Shapiro–Wilk *W* test was applied for normality determination. Student’s *t* test was used for pairwise comparisons and one-way analysis of variance for the effects of skull condition. In the case of absence of interhemispherical differences, the parameters were averaged. Pearson correlation coefficient and linear regression analysis were used to assess associations between changes in parameters, due to IJVC. Differences between correlation coefficients were tested after log transformation. The General Linear Model was used to test for the effect of skull condition on the slopes of linear regressions. A value of *p* < 0.05 was adopted to represent statistical significance. Statistical analysis was performed with STATISTICA (Statsoft Inc., Tulsa, OK).

## Results

We report the inclusion of 98 eligible patients admitted between August 2017 and May 2020. ICP recordings were of poor quality for nine patients. Data of other four patients were lost, yielding a final sample of 85 patients for analyses. Sixteen patients (19%) had intracranial hypertension (baseline ICP > 20 mm Hg). Right and left hemisphere values of CBv, CrCP, and RAP were averaged following pairwise testing. Table 1 presents patient characteristics classified according to the determined groups. ICP was higher (*p* = 0.025), and heart rate was lower (*p* = 0.045) for the group Sk3, but all other variables did not show any significant differences due to skull status.

**Table 1:** Patient characteristics according to injury severity

Variable	All patients (*n* = 85)	Groups			*p* value
		Sk1 (*n* = 24)	Sk2 (*n* = 37)	Sk3 (*n* = 24)	
Age (years)	37.7 ± 21.2	34.2 ± 24.2	37.3 ± 20.9	40.9 ± 20.5	0.57
Male sex	56 (65.9)	14 (58.3)	24 (64.9)	17 (70.8)	0.66
Admission GCS	5.2 ± 3.7	5.0 ± 3.5	5.2 ± 3.6	5.4 ± 4.3	0.96
MAP (mm Hg)	86.7 ± 12.0	89.8 ± 11.8	85.2 ± 11.8	86.7 ± 9.1	0.29
ICP (mm Hg)	15.5 ± 7.6	13.5 ± 6.2	14.6 ± 6.2	19.0 ± 9.7	0.025
CPP (mm Hg)	71.2 ± 13.2	76.3 ± 13.0	70.5 ± 13.5	67.7 ± 11.6	0.064
CBFV (cm × s^−1^)	75.9 ± 23.7	75.7 ± 21.4	78.7 ± 24.0	71.0 ± 25.5	0.47
Heart rate (bpm)	82.4 ± 19.6	82.5 ± 17.9	86.9 ± 18.7	74.3 ± 20.4	0.045
EtCO_2_ baseline (mm Hg)	34.3 ± 4.2	34.5 ± 4.0	34.3 ± 4.4	33.9 ± 4.4	0.90
EtCO_2_ IJVC (mm Hg)	34.1 ± 4.1	34.5 ± 3.9	33.8 ± 4.4	34.3 ± 4	0.13
SO_2_ (%)	98.1 ± 2.3	97.8 ± 2.3	98.1 ± 2.2	98.1 ± 2.5	0.85
CrCP (mmHg)	22.3 ± 13.0	23.2 ± 11.4	21.2 ± 31.0	23.3 ± 15.0	0.77
RAP (mm Hg × s × cm^−1^)	0.96 ± 0.41	0.99 ± 0.45	0.91 ± 0.34	1.03 ± 0.45	0.50
Diagnosis					
TBI	61 (71)	18 (29)	28 (45)	15 (24)	
SAH	15 (17)	5 (33)	6 (40)	4 (26)	
Ischemic stroke	5 (5)	0	0	5 (100)	
AVM	1 (1)	0	1 (100)	0	
Neoplasm	1 (1)	0	1 (100)	0	
IPH	2 (2)	1 (50)	1 (50)	0	

Values are mean ± SD or occurrences (%)

AVM, arterial-venous malformation; CBFv, cerebral blood flow velocity; CPP, cerebral perfusion pressure; CrCP, critical closing pressure; EtCO_2_, end-tidal carbon dioxide; GCS, Glasgow Coma Score; ICP, intracranial pressure; IJVC, internal jugular veins compression; IPH, intraparenchymal hemorrhage; MAP, mean arterial blood pressure; RAP, resistance-area product; SAH, subarachnoid hemorrhage; Sk1, no surgical skull opening; Sk2, craniotomy; Sk3, decompressive craniectomy; SO_2_, oxygen saturation; TBI, traumatic brain injury

### Effects of Internal Jugular Veins Compression

**Fig. 2:**
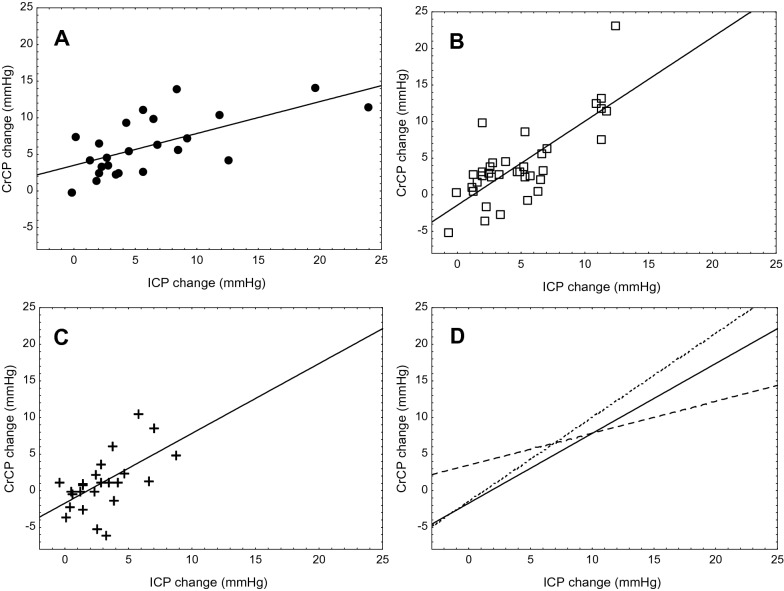
Change in critical closing pressure (CrCP) as a function of the change in intracranial pressure (ICP) resulting from temporary compression of the internal jugular vein. Linear regression lines with raw data correspond to patients with **a** undamaged skull (circles, *r* = 0.643, *p* = 0.0007), **b** craniotomy (open squares, *r* = 0.762, *p* < 0.0001), and **c** craniectomy (crosses, *r* = 0.580, *p* = 0.003). **d** Comparison of regression lines corresponding to undamaged skull (dashed line), craniotomies (dotted line), and craniectomy (solid line)

IJVC led to an increase in ICP and CrCP with a drop in CBv and CPP in all groups. RAP did not have significant changes for groups Sk1 and Sk2, but for group Sk3 there was a marked increase of this parameter. Likewise, MAP response was also remarkable in this group (Fig. 1). The response to compression was not uniform and varied according to injury severity (Table 2). With compression, ΔMAP, ΔCPP, ΔCrCP, and ΔRAP were different between groups, but ΔICP was only borderline (*p* = 0.052). On the other hand, following compression, there was a very strong linear relationship between ΔICP and the corresponding ΔCrCP (Fig. 2), with correlation coefficients of *r* = 0.643 (*p* = 0.0007), *r* = 0.732 (*p* < 0.0001), and *r* = 0.580 (*p* = 0.003) for Sk1, Sk2, and Sk3, respectively. General Linear Model analysis indicated that the three slopes in Fig. 2d were significantly different (*p* = 0.041). Tukey’s post hoc analysis showed differences in slope between Sk3 and both Sk1 (*p* = 0.00012) and Sk2 (*p* = 0.0066). Considering the entire sample (*n* = 85), a strong relationship between absolute values of CrCP and ICP was observed, either before (*p* = 0.0007) or after (*p* = 0.00006) IJVC, as expressed by Pearson’s correlation coefficient.

**Table 2:** Changes in main variables following IJVC according to injury severity

Variable	All patients (*n* = 85)	Groups			*p* value
		Sk1 (*n* = 24)	Sk2 (*n* = 37)	Sk3 (*n* = 24)	
Δ MAP (mm Hg)	1.08 ± 4.73	0.14 ± 3.24	0.17 ± 3.36	3.52 ± 6.87	**0.034**
Δ ICP (mm Hg)	5.41 ± 4.46	6.83 ± 6.14	5.69 ± 3.66	3.39 ± 2.41	0.052
Δ CPP (mm Hg)	− 4.33 ± 6.51	− 6.69 ± 6.59	− 5.51 ± 4.64	0.13 ± 7.02	**0.002**
Δ CBv (cm × s^−1^)	− 5.93 ± 8.28	− 7.06 ± 5.71	− 6.28 ± 9.50	− 4.14 ± 8.78	0.54
Δ Heart rate (bpm)	0.58 ± 2.80	− 0.54 ± 2.53	0.82 ± 2.74	1.44 ± 2.92	0.075
Δ CrCP (mm Hg)	4.54 ± 5.03	6.43 ± 4.42	5.10 ± 5.29	1.57 ± 4.08	**0.007**
Δ RAP (mm Hg cm × s^−1^)	0.03 ± 0.13	− 0.01 ± 0.11	− 0.00 ± 0.10	0.11 ± 0.17	**0.005**

Values are mean ± SD

ΔCBv, change in cerebral blood velocity; ΔCPP, change in cerebral perfusion pressure; ΔCrCP, change in critical closing pressure; ΔICP, change in intracranial pressure; ΔMAP, change in mean arterial blood pressure; ΔRAP, change in resistance-area product; IJVC, internal jugular veins compression; SD, standard deviation; Sk1, no surgical skull opening; Sk2, craniotomy; Sk3, decompressive craniectomy

Tukey’s post hoc test for paired comparisons with sk3 *p* < 0.05 or *p* < 0.005

Bold values are statistically significant

During IJVC, ICP elevation was noted for patients belonging to group Sk1, with the peak of mean ICP values approximately at 20 s of compression onset, followed by a ponderous decrease toward the end of compression. Moreover, after IJVC release, ICP remained lower than baseline for approximately 30 s (Fig. 1c). Similar behavior was not verified for groups Sk2 and Sk3.

### Focal Injury

**Fig. 3:**
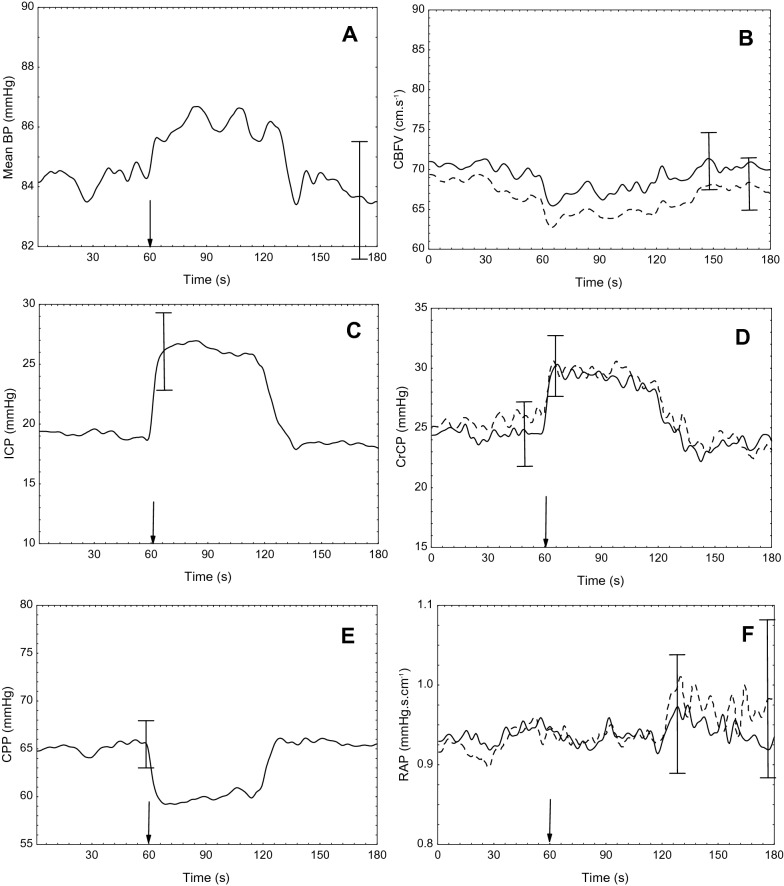
Population average (*n* = 37) of cerebral hemodynamic parameters following temporary compression of the internal jugular vein in patients with unilateral traumatic brain injury. The vertical arrow marks the beginning of compression. **a** Mean arterial blood pressure (MABP); **b** cerebral blood flow velocity (CBFV); **c** intracranial pressure (ICP); **d** critical closing pressure (CrCP); **e** cerebral perfusion pressure (CPP) (CPP = MABP − ICP); and **f** resistance-area product (RAP). Continuous line indicates measurements from the affected hemisphere, dashed line from the nonaffected hemisphere. The error bars represent ± 1 SE at the time of occurrence. SE, standard error of the mean

Subanalysis of patients with a clear tomographic focal injury was performed (*n* = 37). No significant differences were observed for the comparison between injured and noninjured hemispheres for all parameters variations (ΔCBv, ΔCrCP, and ΔRAP) during IJVC (Fig. 3 and Sup. Table 1).

### Effects of Age

For the parameters in Table 1, age was only correlated with ICP (*r* =  − 0.21, *p* = 0.048) and RAP (*r* = 0.40, *p* = 0.00016). No significant correlations with age were observed for any of the parameters in Table 2. The potential interference of age on the main results of the study was also assessed by removing study participants under 18-years-old and repeating the analyses for the adult study participants only. Overall, the results described previously remained the same. The linear regressions between ΔCrCP and ΔICP were not altered, with similar correlation coefficient values and a significant difference between linear regression slopes (*p* = 0.028). For the parameters in Table 2, only ΔHR changed by becoming significant (*p* = 0.028). All other parameters remained significant or nonsignificant as in Table 2.

## Discussion

**Fig. 4:**
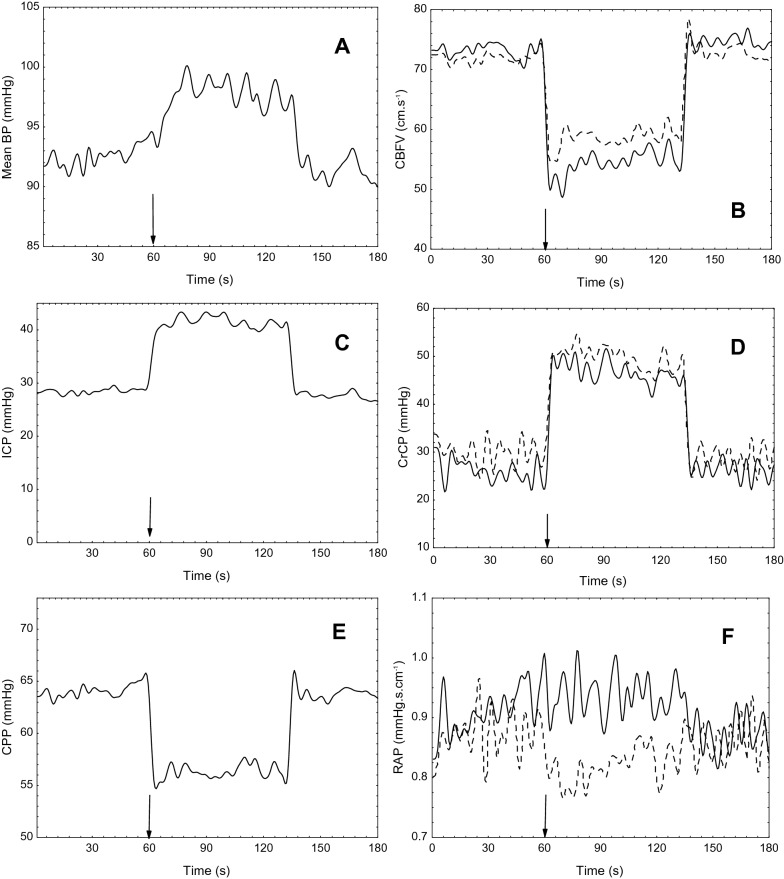
Representative changes in cerebral hemodynamic parameters in a 41-year-old male patient with right traumatic craniotomy for hematoma evacuation. The vertical arrow marks the beginning of jugular veins compression. **a** Mean arterial blood pressure (MABP); **b** cerebral blood flow velocity (CBFV); **c** intracranial pressure (ICP); **d** critical closing pressure (CrCP); **e** cerebral perfusion pressure (CPP = MABP − ICP); and **f** resistance-area product (RAP). Continuous line indicates measurements from the right hemisphere, dashed line from the left hemisphere. ICP was high at the baseline (~ 28 mm Hg) but positively compensated with MABP elevation during compression, leading CPP to not drop lower than 55 mm Hg. CBFV dropped bilaterally but remained under standard values. CrCP elevation was uniform for both hemispheres, whereas RAP elevation was sensitive for the affected (right) hemisphere. BP, blood pressure

The present study has indicated that the cerebral hemodynamic responses following ICP variations can behave differently among patients in the early stages after ABI, according to the severity of these brain injuries. This was markedly observed among patients who presented with refractory raised ICP and underwent DC. To our knowledge, changes in TCD-derived CrCP and RAP using controlled ICP variations have not been previously reported in this population, eliciting a better understanding of the hemodynamic consequences of ABI. Of considerable relevance, the strong linear association between the changes in CrCP and ICP should stimulate further advances in noninvasive methods for assessment of cerebrovascular function (Fig. 4).

### Relevance of CrCP and RAP

In the literature, there is an index also called RAP, however, it is derived from ICP pulse amplitude and ICP mean values. This index is estimated for the assessment of intracranial compensatory reserve [21, 22]. Differently, the RAP parameter used in the current study expressed the concept of CVR, considering that blood velocities assessed with TCD suffer the impact of changes on the vessels cross-sectional area. Therefore, the present study evaluated the impact of induced ICP elevation over small vessels and capillary resistance and the contribution of MAP response to keep CPP stable. It is possible to conclude that cerebrovascular physiology is considerably altered after ABI. However, more work is needed to fully understand the causal pathways involved.

### Practical Applications

Several investigators have proposed that CrCP could replace ICP in noninvasive estimates of CPP [7, 15, 23–26]. Highly significant linear correlations between CrCP and ICP were reported by Thees et al. [15] (*r* = 0.91) in 70 patients and by Czosnyka et al. [24] (*r* = 0.51) in 98 patients with head injury. In our study, we confirmed this strong association between CrCP and ICP when the entire sample was pooled into the same linear regression, as well as for all three distinct groups separately.

The possibility of obtaining optimal CPP estimation, instead of tethering to fixed ICP thresholds [6, 7, 27], is supported by the close similarity of temporal patterns observed for the ICP and CrCP changes following IJVC and for mathematical modeling showing a linear dependence between CrCP and ICP [7]. Interesting, for Sk1, the slope of the linear regression between ΔCrCP and ΔICP is in good approximation to the slope indicated by mathematical modeling [7].

This reinforces the idea that CrCP could be used as a noninvasive surrogate of ICP. However, it should be emphasized that CrCP is influenced by CO_2_ changes [18, 28–31], whereas RAP could be more sensitive to transmural pressure changes in large arteries and arterioles, reflecting their specific myogenic responses to changes in ABP [7, 16, 18, 29, 32–35]. Our results could not entirely exclude some influence of CO_2_ on CrCP findings, which strengthen the importance of very close monitoring of this variable.

From this perspective, cerebral hemodynamic responses of small arterioles and capillaries can be assessed according to ABP, ICP, pCO_2_, and pO_2_ changes. The information provided by CrCP and RAP can add to the beat-to-beat values of mean CBv obtained from TCD and the associations that we and others have reported warrant further investigation in larger studies of ABI, as well as in other cerebrovascular conditions.

### Compensatory Mechanisms

After severe trauma and surgical manipulation of the skull, the loss of natural brain architecture may lead to CSF [36] and venous blood [37, 38] transit impairment. Therefore, the buffering mechanisms of the compensatory reserve, such as CSF displacement toward cervical cisterns and large venous sinuses emptying their volumes to extracranial veins are limited [22, 39]. In our results, this idea was evident because of the distinct ICP behavior between uninjured/not manipulated skulls and the other groups, with ICP beginning to decrease after 20 s of IJVC (Fig. 1c). Otherwise, the plateau during the 60 s of IJVC in cases of craniectomies and craniotomies was sustained.

Recent studies have shown that craniectomized patients may present substantial alterations in intracranial compliance (ICC) (ICC = intracranial volume/pressure) [40], despite consistent drops in ICP values. After DC, we expect an increase in cerebral blood perfusion, possibly due to a lower influence of the rigid cranial vault [38, 41, 42]. On the other hand, TCD and CT perfusion studies reported a wide variety of hemodynamic changes after DC [43–45], with normalization of cerebral blood transit not being sufficient for the determination of outcomes in ABI [46, 47].

Our data demonstrated a very close relationship of RAP and MAP in DC group (Table 2), which could indicate the presence of a myogenic response, even with a considerable degree of CA impairment [48], whereas the reduced ΔCrCP could reflect the depressed CA, with less active wall tension resulting from metabolic pathways [49]. Therefore, after DC—despite the ICP control and often the cerebral perfusion increasing due to brain swelling and stretching, as well as the compensatory reserve and CA impairment—ICC may remain compromised [43, 47]. That is, the “war against ICC impairment is not won yet.” This was also observed by Brasil et al. [41] assessing the ICP waveform slopes in the same stage after injury, supported with the fact that brain hemodynamics often improve with cranioplasty [38, 50].

All this reinforces the idea that CA (or cerebral blood flow regulation) is not an all-or-nothing phenomenon, and the decomposition of CVR in two variables (CrCP and RAP) would give a more realistic bedside interpretation of the complex changes in cerebral hemodynamics of neurocritical patients. Likewise, these findings also emphasize the importance of not considering CPP strictly determined by the interaction between MAP and ICP in ABI [48, 51, 52] and advise for an individualized approach using parameters more representative of the real changes in brain hemodynamics, such as CrCP and RAP.

### Limitations of the Study

The given observations of the present study were supported by the analysis of data extracted from the early stages of ABI in a single monitoring session. It is acknowledged that a cohort study design would increase the possibility to follow-up physiological changes in this population. Our results are derived from MCA blood transit velocity, instead of actual cerebral blood volume or brain perfusion, and this limitation could have affected our results. IJVC increased ICP and this could have caused a reduction in MCA diameter, thus leading to overestimation of the corresponding drop in CBv, as well as small errors in CrCP and RAP. Despite a large range of ages included, our main findings persisted even with the removal of data from patients under 18 years old. Automated and continuous PaCO_2_ was not registered, although maintained within controlled limits due to mechanical ventilation and did not show significant changes during monitoring sessions; rather, EtCO_2_ was used as a proxy of changes in arterial CO_2_ tension.

To maintain the focus of the study on the main hypothesis outlined in the Introduction, we have not explored other interparameter relationships, such as the association between ΔCrCP and ΔRAP. Changes in RAP due to IJVC and its association with corresponding changes in MAP can reflect the status of dynamic CA, which are under investigation and will be reported elsewhere. Finally, TCD measurements were performed exclusively on main distal branches of internal carotid arteries, the MCAs more specifically, not considering disturbances on the posterior fossa.

## Conclusions

Critical closing pressure is correlated with ICP variations and may aid in planning therapeutic interventions based on hemodynamic adjustments. An induced mild ICP elevation in the present study did not impact the CPP of craniectomized patients. These patients demonstrated elevated MAP response to ICP variation, despite the persistence of higher CVR, which may be interpreted as a protection mechanism provided by DC. Patients with ABI not submitted to neurosurgical procedures revealed a higher capacity of handling artificial ICP elevation, which may be attributed to less severe injury, appropriate ICC, and efficient compensatory reserve. These observations altogether reinforce the need for multimodal monitoring of the neurocritical patient.

### Supplementary Information

Below is the link to the electronic supplementary material.

Supplementary file1 (DOCX 34 kb)

Supplementary file2 (DOCX 12 kb)

## Clinical Trial Registration

Trial registration: NCT03144219, Registered 01 May 2017 Retrospectively registered, https://www.clinicaltrials.gov/ct2/show/NCT03144219

ClinicalTrials.gov: NCT03694678.
