# Supplementary material for: Critical Closing Pressure and Cerebrovascular Resistance Responses to Intracranial Pressure Variations in Neurocritical Patients
Source: Neurocrit Care. 2023 Mar 3;39(2):399–410. doi: 10.1007/s12028-023-01691-8 (PMC10541829; doi:10.1007/s12028-023-01691-8)
Supplement: Supplementary file 1 — Supplementary file1 (DOCX 34 kb) [file 12028_2023_1691_MOESM1_ESM.docx]

Supplemental figure – Population average (n=39) of cerebral hemodynamic parameters following temporary compression of the internal jugular vein in patients with bilateral or diffuse traumatic brain injury. The vertical arrow marks the beginning of compression. (A) Mean arterial blood pressure (MABP); (B) Cerebral blood flow velocity (CBFV); (C) Intracranial pressure (ICP); (D) Critical closing pressure (CrCP); (E) Cerebral perfusion pressure (CPP=MABP-ICP); and (F) Resistance-area product (RAP). Continuous line indicates measurements from the right MCA, dashed line from the left MCA. To facilitate visual comparison, the y-axis scales have been kept the same as in Fig. 2. The error bars represent ± 1 SE at the time of occurrence.
