# Supplementary material for: Critical Closing Pressure and Cerebrovascular Resistance Responses to Intracranial Pressure Variations in Neurocritical Patients
Source: Neurocrit Care. 2023 Mar 3;39(2):399–410. doi: 10.1007/s12028-023-01691-8 (PMC10541829; doi:10.1007/s12028-023-01691-8)
Supplement: Supplementary file 2 — Supplementary file2 (DOCX 12 kb) [file 12028_2023_1691_MOESM2_ESM.docx]

| Variable | affected hemisphere | NAH | p-value |
| --- | --- | --- | --- |
| ΔCBv (cm/s) | -2.63 ± 5.92 | -2.71 ± 4.87 | 0.91 |
| ΔCrCP (mmHg) | 4.82 ± 5.29 | 4.10 ± 5.44 | 0.25 |
| ΔRAP (mmHg.s/cm) | -0.01 ± 0.12 | -0.005 ± 0.10 | 0.66 |

Sup. Table 1. Comparison pre- and post-compression between affected hemisphere (n=37) and non-affected hemisphere for focal injuries. Δ: difference from prior (baseline) and after 30 seconds of jugular compression.
